# Role of Leptin/Osteopontin Axis in the Function of Eosinophils in Allergic Rhinitis with Obesity

**DOI:** 10.1155/2018/9138904

**Published:** 2018-10-24

**Authors:** Wenlong Liu, Qingxiang Zeng, Yanqiu Chen, Ren Zhong Luo

**Affiliations:** Department of Otolaryngology, Guangzhou Women and Children's Medical Center, Guangzhou Medical University, Guangzhou, China

## Abstract

**Background:**

Allergic rhinitis (AR) is characterized by tissue and blood eosinophilia. Previous studies showed enhanced eosinophilia in allergic rhinitis patients with obesity, suggesting an association between obesity and eosinophilia. However, the interaction and mechanism between obesity and eosinophilia is still unclear.

**Methods:**

We recruited thirty AR children and 30 controls in this study. Expression of leptin and osteopontin (OPN) proteins in serum was detected, and correlation analysis with eosinophilia was performed. The effect of leptin or OPN on eosinophil apoptosis, adhesion, migration, and activation of eosinophil was examined. Ovalbumin-sensitized mice were established to prove the role of obesity on eosinophil regulation by leptin and OPN.

**Results:**

We found that upregulated serum and nasal leptin and OPN expression in AR were positively correlated with eosinophilia and eosinophil cationic protein levels. Leptin or OPN inhibited eosinophil apoptosis, demonstrated as inhibited DNA fragmentation and phosphatidylserine (PS) redistribution (*P* < 0.05). Leptin and OPN promote expression of cluster of differentiation 18 (CD-18) and intercellular adhesion molecule 1 (ICAM-1) and inhibit expression of ICAM-1 and L-selectin by eosinophils, which contribute to the adhesion of eosinophils. Leptin and OPN mediated migration and activation of eosinophil through phosphatidylinositol-3-OH kinase (PI3K) pathway. Obese AR mice presented with more severe eosinophilia and symptoms compared with nonobese AR mice or control mice. Immunochemistry staining of leptin and OPN of nasal turbinate in obese AR mice was also stronger than those in nonobese AR mice or control mice. Anti-OPN, anti-leptin, and anti-*α*4 treatments reduce nasal eosinophilia inflammation and clinical symptoms in model mice.

**Conclusion:**

Our results suggested that in an obese state, upregulation of leptin and OPN regulates apoptosis, adhesion, migration, and activation of eosinophils, and this process may be mediated by the PI3K and anti-*α*4 pathways.

## 1. Introduction

Tissue and blood eosinophilia is a common feature in allergic airway diseases such as allergic rhinitis (AR), nasal polyposis, and bronchial asthma [1]. Delayed eosinophil apoptosis contributes to eosinophilia, and the infiltration and subsequent activation of eosinophils in the airways result in the secretion of specific granule proteins, synthesis, and release of lipid mediators, inflammatory cytokines, chemokines, and growth factors [2, 3]. Through these mediators, eosinophils contribute to the perpetuation and amplification of airway inflammation [4]. Previous studies have shown an association between obesity and eosinophilia [5, 6]. However, the interaction and mechanism between obesity and eosinophilia are still unclear.

Leptin, an adipokine of the obesity (ob) gene, has been shown to regulate food intake and energy expenditure via a hypothalamic-mediated effect [7, 8]. Leptin's functional receptor (ObRb) is expressed in the hypothalamus and various immune cells including T and B lymphocytes, natural killer cells, monocytes, and eosinophils [9, 10]. Previous study showed that serum leptin level is related to body fat percentage and body fat mass [11]. Most recently, Conus et al. reported that human eosinophils can express Ob-Rb and delay spontaneous eosinophil apoptosis [12]. Besides, leptin has been shown to upregulate surface adhesion molecules, induce the release of inflammatory cytokines, and stimulate migration of eosinophils [13].

Osteopontin (OPN) is an extracellular matrix protein with a wide range of functions. It contains arginine-glycine-aspartate (RGD) and SVVYGLR domains that bind various integrins and promote adhesion and migration of inflammatory cells [14, 15]. Osteopontin is also expressed in human eosinophils and is increased after granulocyte-macrophage colony-stimulating factor (GM-CSF) and IL-5 activation [16]. Besides, recombinant OPN promotes eosinophil chemotaxis in vitro, and this effect is mediated by *α*4*β*1 integrin binding [16].

In view of the similar roles of OPN and leptin in the regulation of eosinophil, we postulated that there may be an interplay between OPN and leptin in AR. In this study, we aimed to explore the correlation between leptin and OPN in eosinophilia of AR children with obesity.

## 2. Materials and Methods

### 2.1. Patients

We recruited thirty AR children (5–10 years old) from the Department of Otolaryngology, Guangzhou Women and Children's Medical Center, from January 2016 to October 2016. Local ethics committee boards approved the study and informed consent was signed by the parents. Perennial AR was diagnosed according to disease history, nasal examination, and specific IgE values according to the Allergic Rhinitis and its Impact on Asthma (ARIA) guideline (2010) [17]. The common inhalant allergens (dust mites, pets, molds, cockroach, etc.) were assessed by the measurement of specific IgE (Phadia AB, Uppsala, Sweden) with values greater than or equal to 0.35 kIU/L as positive. Thirty healthy children (5–10 years old) were recruited as controls.

Children with other allergic diseases (atopic dermatitis, allergic asthma, etc.), purulent nasal infection, any infection within the past 2 weeks, and recent use of drugs (systemic or topical corticosteroids, sodium cromoglycate, and histamine H1 antagonist) were excluded from this study.

Body weight and height were measured using an electronic weighing scale, and body mass index (BMI) was calculated. BMIs greater than the 95th percentile were considered as obesity according to a local population survey [18].

### 2.2. Symptom Scores

Nasal symptoms including sneezing, rhinorrhea, itchy nose, and nasal congestion were recorded and assessed as follows: 0, none; 1 point, mild; 2 points, moderate; and 3 points, severe. The total nasal symptom score (TNSS) was calculated accordingly. The nasal symptoms were averaged in an 8-week observation period.

### 2.3. Blood Sample Collection

Fasting venous blood samples were collected between 6 am and 8 am. The samples were centrifuged at 1000g for 15 minutes at 4°C and stored at −80°C for further measurement.

### 2.4. ELISA and Eosinophil Cationic Protein (ECP) Detection

The serum levels of leptin and OPN were measured in duplicate and averaged using ELISA kits (R&D, Minneapolis, USA) according to the manufacturer's instructions. Total serum IgE level was tested by an ELX-800 system. ECP level was detected using Unicap system (Phadia AB, Uppsala, Sweden). The detection limits of the assays were as follows: leptin (22 pg/mL), OPN (312.5 pg/mL), and ECP (2.0 ng/mL). Results were from three independent experiments.

### 2.5. Isolation of Human Blood Eosinophils from Buffy Coat and Eosinophil Culture

Human eosinophils were purified from the venous blood of children by MACS-negative immunomagnetic isolation kit (Miltenyi Biotec, Bergisch Gladbach, Germany) according to the instructions provided by the manufacturer. The purity of eosinophils was 98–100% (Giemsa staining) and the viability was larger than 98% (Trypan blue staining). The isolated eosinophils were cultured in RPMI 1640 medium supplemented with 10% FBS and 20 mM HEPES (Gibco, New York, USA).

### 2.6. Leptin and OPN Delay Cell Death and Apoptosis and Induce Adhesion, Migration, and Activation of Eosinophil

Eosinophil death was assessed based on uptake of 1 mM ethidium bromide and flow cytometric analysis (FACS Calibur, BD, New Jersey, USA) described as previously [12]. DNA fragmentation and redistribution of phosphatidylserine (PS) were also detected to determine whether cell death was apoptosis as previously described [12]. Oligonucleosomal DNA fragmentation, characteristics of apoptosis, was assessed by flow cytometry. In brief, eosinophils suspended in 0.3 mL hypotonic fluorochrome solution (50 *μ*g/mL propidium iodide, 0.1% sodium citrate, 0.1% Triton X-100) for 10 hours in the dark at 4°C. Then, the relative amounts of apoptotic eosinophils were determined by discrimination of hypodiploid and diploid cells through flow cytometry. PS appears on the external leaflet in apoptotic cells, and Annexin V is a PS-binding protein used for detecting apoptotic cells. Therefore, a commercial apoptosis detection kit (R&D, Minneapolis, USA) was used for evaluating the appearance of PS assessed by flow cytometry. All results were from three independent experiments.

In the adhesion assay, 96-well plates (Maxisorp, Nunc, Roskilde, Denmark) were prepared by coating individual wells with 60 *μ*L of fibronectin overnight at 4°C. The eosinophil suspension was then incubated for 30 minutes at 37°C (5% CO_2_) with different concentration of leptin or OPN. After incubation of treated cells, the nonadhered cells were removed, and the remaining cells were washed twice with PBS. The eosinophil adhesion was calculated by measuring the residual eosinophil peroxidase (EPO) activity of adherent cells as described previously [19]. In brief, EPO substrate (Sigma Chemical, Missouri, USA) was incubated with cell suspension for 30 min at room temperature. After the reaction was terminated by H_2_SO_4_, the absorbance was determined at 490 nm in a microplate reader (Multiscan MS; Labsystems, Helsinki, Finland). The adherence was calculated by comparing the absorbance of the samples to standard curve. Expression of surface adhesion molecule was analyzed using flow cytometry as median fluorescence intensity (MFI). Results were from three independent experiments.

Different concentrations of recombinant OPN (R&D systems, 0.1–1 *μ*g/mL), leptin (R&D systems, 0.1–1 *μ*g/mL), or inhibitors were incubated with eosinophil (10^5^/mL) treated with or without anti-OPN antibody (R&D systems, 1 *μ*g/mL) in 24-well transwell system. Recombinant eotaxin (R&D systems) was used as positive control of chemotaxis analysis. After incubation, the number of migrated cells was determined by Giemsa staining. Migrated cells were counted in 10 fields using light microscopy at a magnification of 400x. ECP level in the supernatant was measured using the Unicap system. Results were from three independent experiments.

### 2.7. Animal Models

Sixty male C57BL6/J mice of four-week-old were raised and fed with standard diet (carbohydrate: 70%; protein: 20%; and fat: 10%) or a high-fat diet (HFD) (carbohydrate: 29%; protein: 16%; and fat: 55%) for ten weeks. The mice were randomly grouped as control (10 mice), OVA (10 mice), OVA + obese (10 mice), and intervention groups (20 mice).

On day 0, all the mice were injected with 100 mg (0.4 mL) of ovalbumin (OVA) (grade V; Sigma-Aldrich, Missouri, USA) mixed with 1.6 mg Al(OH)_3_ in 0.9% NaCl subcutaneously. On day 7, a second subcutaneous injection of 100 mg OVA was given. The control mice received only a subcutaneous injection of Al(OH)_3_. From day 14 to day 18, the mice were challenged nasally by the administration of 10 mg of OVA in 40 mL of PBS (20 mL per nostril) or saline for controls. The mice were anaesthetized and exsanguinated one day after the final challenge, and the peripheral blood was obtained from the abdominal vena cava. Serum total cholesterol (TC), high-density lipoprotein (HDL), and triglycerides (TGs) were tested using commercially available kits. Epididymal fat mass was weighed and nasal turbinate were sampled for morphological study. Nasal symptoms were evaluated by counting the frequency of nasal rubbing and sneezing during a 15-minute observation period.

The 100 *μ*g of recombinant mouse anti-leptin, anti-OPN protein, or anti-*α*4 integrin (R&D Systems, Minneapolis, USA) in 40 mL of PBS was given intranasally 2 hours before each OVA challenge in different subgroups, respectively.

### 2.8. Morphological Study

Nasal turbinate sections (4 to 5 *μ*m) were prepared through deparaffinization xylene and rehydration alcohol. After incubation with 0.3% H_2_O_2_ and pretreated by autoclave heating in citrate buffer (pH 6.0) for 20 minutes, the haematoxylin–eosin (HE) staining was performed. For immunohistochemistry (IHC), the section was treated with rabbit polyclonal antibody against OPN (Zhongshan Golden Bridge, Beijing, China) (1 : 200) or rabbit polyclonal antibody against leptin (Thermo Fisher Scientific, Fremont, CA, USA) overnight at 4°C. Twenty-four hours later, the sections were incubated with secondary biotinylated goat anti-mouse/rabbit IgG antibody and then with avidin-peroxidase complex. Isotype-matched IgG was selected as negative control.

### 2.9. Statistical Analysis

All data are presented as the medians and interquartile ranges. The Kruskal-Wallis H test and the nonparametric Mann–Whitney *U* test were performed using the SPSS21 software. Spearman rank correlation analysis was done for assessment of correlations between various parameters. A *P* value of less than 0.05 was considered as significant.

## 3. Results

### 3.1. Upregulated Serum Leptin and OPN Levels in AR Children and Its Correlation with Eosinophils

**Figure 1:**
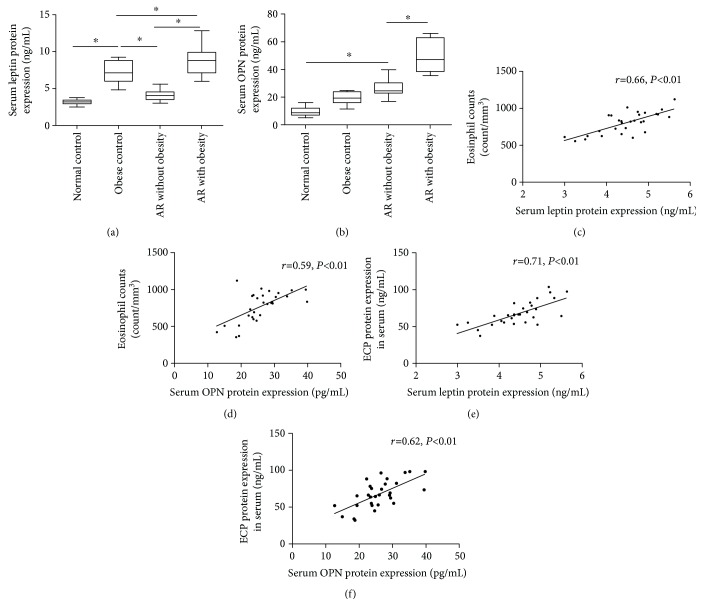
Elevated serum leptin (a) and OPN (b) expression in AR children compared with controls, especially in patients with obesity. (c–f) The positive associations between leptin or OPN and serum eosinophil counts and ECP levels in patients with AR; ^∗^*P* < 0.05. The *N* number of Figures 1(a) and 1(b) is 53 (normal control, 12; obese control, 11; and AR, 30). The *N* number of Figures 1(c)–1(f) is 53 (normal control, 12; obese control, 11; and AR, 30).

The demographic characteristics of all children are summarized in Table 1. The subgroups had comparable age, sex ratio, and age (*P* > 0.05). The AR children with obesity had higher TNSS score compared with AR children without obesity. Our data indicated that serum leptin and OPN levels were significantly higher in AR children compared with controls, especially in obese children (*P* < 0.05) (Figure 1). The upregulated serum leptin and OPN levels were also positively correlated with eosinophil counts (*r* = 0.66, *P* < 0.01; *r* = 0.59, *P* < 0.01) and ECP concentration (*r* = 0.71, *P* < 0.01; *r* = 0.62, *P* < 0.01) in AR children, suggesting that leptin and OPN levels may be involved in eosinophil infiltration and activation (Figure 1).

### 3.2. Leptin/OPN Expression and Function Analysis of Eosinophils

**Figure 2:**
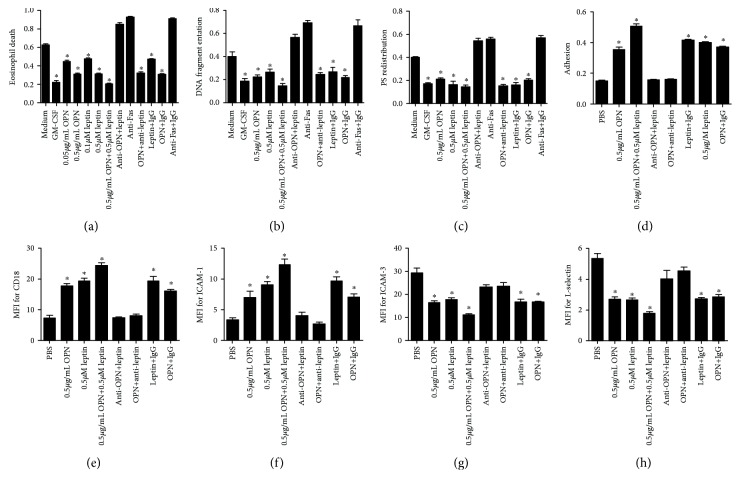
The relative ratio of eosinophil death (a), DNA fragmentation (b), PS distribution (c), and adhesion (d) after leptin and/or OPN stimulation. The expression of adhesion molecules such as CD18 (e), ICAM-1(f), ICAM-3 (g), and L-selectin (h) are shown as MFI. ^∗^*P* < 0.05, compared with the control group.

Leptin and OPN delayed spontaneous eosinophil death in a dose-dependent manner (Figure 2(a)) similar to GM-CSF, and the effect was enhanced when leptin and OPN were given together. Our results showed that leptin reduced DNA fragmentation, which featured by apoptotic eosinophils (Figure 2(b)). We also found that leptin and OPN significantly blocked apoptosis, whereas anti-Fas stimulation resulted in increased PS redistribution (Figure 2(c)). Interestingly, all the above effects were inhibited when leptin and anti-OPN were added, suggesting that the antiapoptotic effect was mainly mediated by OPN.

Leptin and OPN also promote eosinophil adhesion to human fibronectin (Figure 2(d)), and the effect was enhanced when leptin and OPN were given together. Our results also show that either of leptin and OPN induced the surface expression of cluster of differentiation 18 (CD-18) and intercellular adhesion molecule 1 (ICAM-1), while downregulated those of ICAM-3 and L-selectin (Figures 2(e)–2(h)), especially when the two were given together. Interestingly, all the above effects were inhibited when anti-leptin or anti-OPN was added, suggesting that the adhesion effect was mediated by leptin and OPN together.

**Figure 3:**
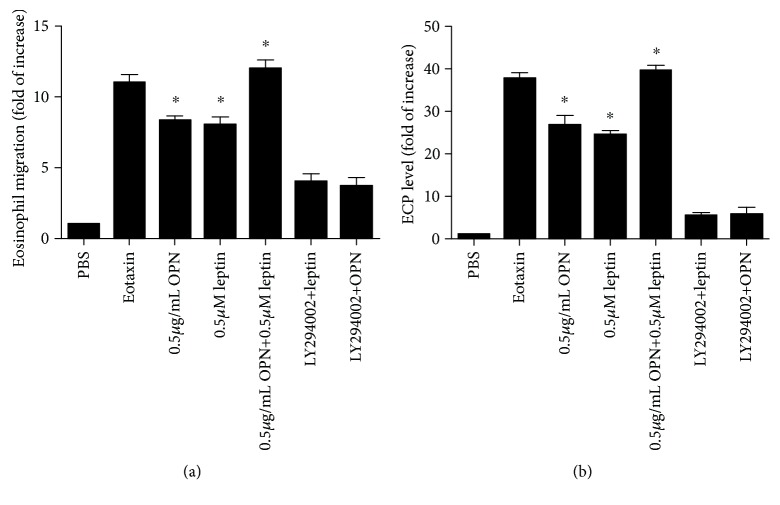
OPN and leptin significantly enhanced EOS chemotaxis (a) and activation (b) after stimulation for 12 h; this effect was enhanced when OPN and leptin were added together and inhibited when LY294002 was added. ^∗^*P* < 0.05, compared with the control group.

To prove the stimulatory effects of leptin on the chemotactic behavior of eosinophils, Transwell analysis was performed. We examined the effects of leptin and OPN on isolated eosinophils from AR patients in a Transwell system *in vitro*. We found that leptin and OPN significantly enhanced eosinophil chemotaxis, as well as activated eosinophils as indicated by increased ECP level, especially when the two were given together (Figures 3(a) and 3(b)). These effects were significantly inhibited by anti-OPN, anti-leptin, and a PI3K specific inhibitor, LY294002, suggesting that leptin and OPN are able to specifically attract and activate eosinophils through PI3K signaling pathway.

### 3.3. HFD Promote Airway Eosinophilic Inflammation and Nasal Leptin/OPN Expression

High-fat diet mice presented with heavier body weight, heavier subcutaneous and visceral fat pad weight, and higher serum levels of TC and LDL compared with controls. The TGs and HDL levels had no differences between groups (Table 2).

**Figure 4:**
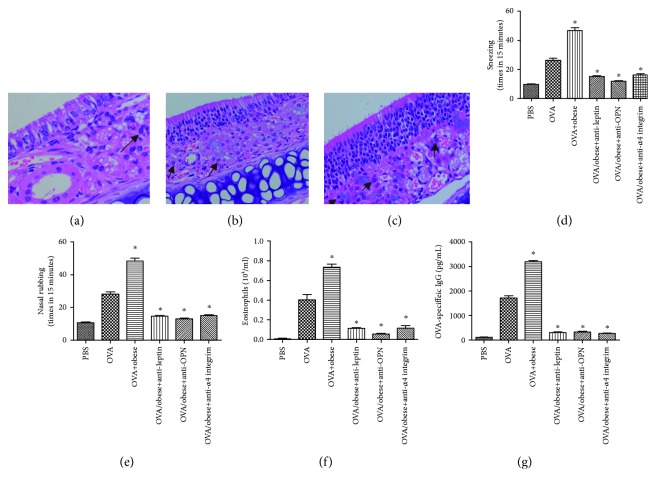
(a–c) HE staining showed that eosinophils in the nasal mucosa of OVA-challenged obese mice were significantly higher than those in OVA-challenged nonobese and control mice. The times of nasal rubbing and sneezing (d, e), the count of eosinophils (f), and OVA-specific IgE (g) were significantly higher in obese AR mice and block of leptin, OPN, or *α*4 integrin alleviate inflammation in obese AR mice. ^∗^*P* < 0.05, compared with the control group. Ten mice were included in each group. The arrow shows representative cells.

HE staining indicated that the number of eosinophils in the nasal mucosa of OVA-challenged obese mice was significantly higher than that of OVA-challenged nonobese and control mice (*P* < 0.05, Figures 4(a)–4(c)). The OVA-specific IgE, the count of eosinophils, and the times of nasal rubbing and sneezing in obese AR mice were significantly higher than those in nonobese AR mice (*P* < 0.05, Figure 4). We also found that the block of leptin, OPN, or *α*4 integrin alleviates inflammation in the models, showed as milder symptoms and eosinophil inflammation (*P* < 0.05).

**Figure 5:**
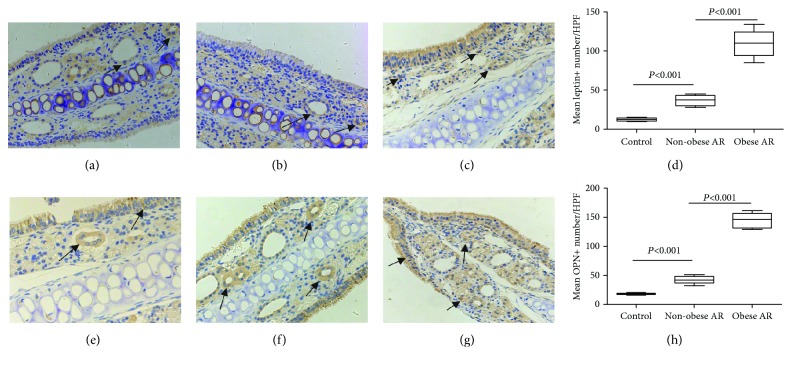
The distribution of leptin and OPN-positive cells in the nasal mucosa of OVA-challenged obese mice (c, g), OVA-challenged nonobese, and control mice (a, e). The count of leptin-positive cells (d) and OPN-positive cells (h) in nasal tissues was significantly higher compared with normal controls (The arrow shows representative cells).

In nasal tissues, IHC staining showed that leptin and OPN-positive cells included epithelial cells, interstitial cells, and glandular cells. The number of OPN and leptin-positive cells per high power field (HPF, 400x) in obese AR mice was significantly higher than that of nonobese AR mice and normal controls (*P* < 0.05, Figure 5).

## 4. Discussion

In the present study, we demonstrated that the expression of OPN and leptin was significantly increased in AR children, especially those with obesity. We also established a clear relationship between apoptosis, adhesion, and activation of eosinophils and leptin/OPN axis. We also provided evidence that *α*4 integrin and PI3K were involved in the process. These results suggested that leptin and OPN may be used as a promising biomarker for eosinophilia in AR patients.

In this study, we first found that upregulation of leptin and OPN in obese children with AR was correlated with the count and activation (ECP level) of eosinophil inflammation compared with nonobese children with AR or controls, suggesting that obesity may be involved in the regulation of eosinophil through leptin and OPN. Leptin is one of the energy-regulating hormones, and serum leptin is positively correlated with body fat mass. Our and previous studies had proved that both leptin and OPN are upregulated during AR and involved in Th2 response. Moreover, it has been reported that serum OPN levels are correlated with obese state and may be reduced by loss of fat mass. Therefore, the elevated level of leptin and OPN in our study was consistent with previous reports [20–23].

Next, we performed *in vitro* experiments using purified eosinophils. Our data showed that leptin and OPN delayed spontaneous eosinophil death in a dose-dependent manner, which is consistent with Conus et al.'s study [12]. We also found that the efficacy of leptin and leptin to block eosinophil apoptosis was similar to that of GM-CSF. Interestingly, when anti-OPN antibody was added, the leptin-mediated antiapoptotic effect on eosinophils was blocked significantly. However, anti-leptin has no obvious effect on OPN-mediated antiapoptotic effect on eosinophils. These results suggested that OPN may play a central role in the process of eosinophil apoptosis, and leptin affects eosinophil apoptosis through the regulation of OPN expression.

As for adhesion to fibronectin, our results showed that leptin and OPN promoted adhesion synergistically and either anti-leptin or anti-OPN can block the adhesion. The interaction of ICAM-1 and integrins has been shown to be essential for the recruitment and trans-endothelial migration of eosinophils [24]. The upregulation of both ICAM-1 and CD18 expressions is therefore essential for the recruitment and transmigration of eosinophils into inflammatory sites [13, 25]. On the other side, ICAM-3 is highly expressed on resting eosinophil surface [26]. Consistently, our results suggested leptin and OPN synergistically promoted ICAM-1 and CD18 and inhibited ICAM-3 expression. In addition, we found that L-selectin expression was also significantly suppressed by leptin and OPN. This finding concurs with several other studies showing that activated eosinophils could downregulate L-selectin [27, 28].

In eosinophil migration assay, we found that both leptin and OPN may promote migration of eosinophils, which was similar to that of eotaxin. Our results suggested that eosinophils preincubated with leptin and/or OPN showed significantly enhanced eosinophil chemotaxis and activation through PI3K pathway, manifested as eosinophil migration and upregulation of ECP. Consistently, Wong et al. [13] also found that leptin-induced eosinophil migration is associated with MAPK activation. Takahash et al. and Puxeddu et al. reported that OPN plays a role in the migration of eosinophils, and it was mediated via interaction between the RGD and SVVYGLR domains on OPN and *α*4 integrin expressed on the surface of eosinophils [16, 29]. These above results suggested that leptin and OPN regulate eosinophil chemotaxis and activation through different pathways.

In the mice model, we found that enhanced eosinophil inflammation and disease severity in obese-OVA mice compared with OVA and control mice. The local expression of leptin/OPN by nasal tissues in obese-OVA mice was also significantly higher compared with that in OVA and control mice. Since the *α*4 integrin has been demonstrated to be highly expressed on eosinophils and primarily responsible for eosinophil trafficking in the airways, reduced nasal inflammation by blockade of *α*4 integrin suggested that leptin and OPN may mediate eosinophil inflammation through *α*4 integrin receptor on OPN [30]. Similarly, the inhibitory effects of anti-*α*4 integrin antibody on the development of allergic airway inflammation have been reported in an animal model [31, 32], and this effect is mediated by the inhibition of migration of eosinophils into the airway [32].

## 5. Conclusions

In summary, our results confirmed that upregulation of leptin and OPN in AR regulates apoptosis, adhesion, migration, and activation of eosinophils, which is mediated by the PI3K and anti-*α*4 pathways. Future studies were needed to prove the detailed interaction between leptin and OPN under obese and allergic state.

## Figures and Tables

**Table 1:** Demographic characteristic of AR children and controls.

Groups	AR without obesity group	AR with obesity group	Obesity control	Control
Number	15	15	15	15
Sex (male : female)	7 : 8	7 : 8	8 : 7	9 : 6
Age (months)	92.1 ± 31.2	87.3 ± 25.6	85.3 ± 27.6	93.5 ± 29.6
BMI	16.6 ± 1.6	24.1 ± 1.8^∗^	23.9 ± 1.6^∗^	16.1 ± 1.5
TNSS score	8.1 ± 2.5	9.9 ± 2.7^#^	—	—
Nasal steroid	10 (67%)	12 (75%)	—	—
Nasal antihistamine	12 (75%)	10 (67%)	—	—
Oral antihistamine	11 (73%)	9 (60%)	—	—

^∗^Compared with control group, *p* < 0.05. ^#^Compared with AR without obesity group, *p* < 0.05.

**Table 2:** Anthropometric and lipid profiles of obese and control mice.

	Control mice	Obese mice
Body weight (g)	24.1 ± 0.23	39.2 ± 0.33^∗^
Epididymal fat (g)	0.28 ± 0.04	1.26 ± 0.06^∗^
TC (mg/L)	912 ± 52	1265 ± 91^∗^
LDL (mg/L)	211 ± 35	537 ± 58^∗^
HDL (mg/L)	501 ± 29	578 ± 36
TGs (mg/L)	711 ± 45	788 ± 53

^∗^Compared with control group, *p* < 0.05.

## Data Availability

The data used to support the findings of this study are available from the corresponding author upon request.
